# Enrichment of phosphate-accumulating organisms (PAOs) in a microfluidic model biofilm system by mimicking a typical aerobic granular sludge feast/famine regime

**DOI:** 10.1007/s00253-022-11759-8

**Published:** 2022-01-15

**Authors:** Edina Klein, Janek Weiler, Michael Wagner, Minja Čelikić, Christof M. Niemeyer, Harald Horn, Johannes Gescher

**Affiliations:** 1 Institute for Technical Microbiology (TMI), Hamburg University of Technology (TUHH), Kasernenstr. 12, 21073 Hamburg, Germany; 2 Institute for Biological Interfaces (IBG-1), Karlsruhe Institute of Technology (KIT), Herrmann-von-Helmholtz Platz 1, 76344 Eggenstein-Leopoldshafen, Germany; 3 Engler-Bunte-Institut (EBI), Water Chemistry and Water Technology, Karlsruhe Institute of Technology (KIT), Engler-Bunte-Ring 9a, 76131 Karlsruhe, Germany

**Keywords:** Biofilms, Wastewater treatment, Polyphosphate-accumulating organisms (PAOs), Microfluidic, Aerobic granular sludge (AGS), Enhanced biological phosphorus removal (EBPR)

## Abstract

Wastewater treatment using aerobic granular sludge has gained increasing interest due to its advantages compared to conventional activated sludge. The technology allows simultaneous removal of organic carbon, nitrogen, and phosphorus in a single reactor system and is independent of space-intensive settling tanks. However, due to the microscale, an analysis of processes and microbial population along the radius of granules is challenging. Here, we introduce a model system for aerobic granular sludge on a small scale by using a machine-assisted microfluidic cultivation platform. With an implemented logic module that controls solenoid valves, we realized alternating oxic hunger and anoxic feeding phases for the biofilms growing within. Sampling during ongoing anoxic cultivation directly from the cultivation channel was achieved with a robotic sampling device. Analysis of the biofilms was conducted using optical coherence tomography, fluorescence in situ hybridization, and amplicon sequencing. Using this setup, it was possible to significantly enrich the percentage of polyphosphate-accumulating organisms (PAO) belonging to the family *Rhodocyclaceae* in the community compared to the starting inoculum. With the aid of this miniature model system, it is now possible to investigate the influence of a multitude of process parameters in a highly parallel way to understand and efficiently optimize aerobic granular sludge-based wastewater treatment systems.

**Key points**

• *Development of a microfluidic model to study EBPR.*

• *Feast-famine regime enriches polyphosphate-accumulating organisms (PAOs).*

• *Microfluidics replace sequencing batch reactors for aerobic granular sludge research.*

**Supplementary Information:**

The online version contains supplementary material available at 10.1007/s00253-022-11759-8.

## Introduction

The activated sludge system is the most common method for conventional wastewater treatment. Still, it is space demanding, which is a major obstacle in the context of a growing world population and simultaneous urbanization. Therefore, more compact systems for wastewater treatment are preferable. Due to the poor settling velocity of activated sludge flocs, most of the space required for conventional treatment systems is needed for settling tanks. A particularly promising alternative is the utilization of aerobic granular sludge (AGS) instead of activated sludge flocs (Morgenroth et al. 1997; de Bruin et al. 2004; Adav et al. 2008). Increased settling velocities can be achieved due to the physical characteristics of the self-aggregating granules (Zheng et al. 2005; Zheng et al. 2006; Shi et al. 2009; Xiao et al. 2008). For this reason, biological treatment and biomass separation are possible in one reactor, so that space-intensive secondary clarifiers and pumping costs for recirculation sludge can be avoided (Sarma and Tay 2018). The biomass is stimulated to form compact granules by adjusting specific process parameters. Granular sludge biomass is developed in sequencing batch reactors (SBRs). Among other factors, high shear forces in combination with a short settling phase are required to select for fast-sinking granules in contrast to sludge flocs (de Bruin et al. 2004; Beun et al. 2002; Tay et al. 2001). The use of alternating cycles of oxic hunger phases and anoxic feeding phases was identified as a further important process factor, which leads to an improved granular stability and higher nitrogen and phosphorus elimination rates (de Kreuk et al. 2004; Wang et al. 2018). Within the anoxic phase, easy to metabolize compounds are converted into storage polymers like polyhydroxyalkanoates (PHAs). The limited energy availability due to a lack of electron acceptor during this phase leads to an overall reduced growth rate of heterotrophic bacteria and the development of a niche for slow but resource-efficient growing microorganisms (Krishna and van Loosdrecht 1999; van Loosdrecht et al. 2006). Furthermore, the formation of storage polymers within the feast-famine-regime increases biomass density (Oshiki et al. 2010) and supports biological phosphorus removal (de Kreuk and van Loosdrecht 2004). The required energy for PHA globule production in the anoxic feast phase is primarily obtained by degrading intracellular stored polyphosphate (Poly-P), which is released as orthophosphate into the surrounding medium. In the subsequent oxic phase, these reactions are reversed. Using the stored PHAs as an energy source, phosphate is taken up from the surrounding medium to replenish the Poly-P storage. Due to an increased uptake of phosphate in the oxic phase and sludge removal at this time compared to the phosphate release in the anoxic phase a net elimination of phosphorus occurs (Pijuan et al. 2006). Besides these externally employed temporal variations of oxygen concentrations, each granule itself is characterized by a steep oxygen diffusion gradient from the surface towards the center. Hence, even during the oxic phase, anaerobic metabolic processes can take place within the particles (Kishida et al. 2006; Kagawa et al. 2015). Thus, nitrogen elimination is possible via nitrifying organisms in the outer oxygen penetrated layer and denitrifying organisms in the oxygen-free inner space of the particles. Phosphate-accumulating organisms (PAOs), for which the internally stored polymers serve as storage substances as described above, can be found in all layers of the granules (Kagawa et al. 2015). Since PAOs can use these storage substances as electron donors for denitrification, the organisms often fulfill a dual function (phosphate removal and denitrification; Lee and Yun 2014).

Although domestic wastewater is already treated in aerobic, granule-based large-scale sewage treatment plants, a detailed investigation of processes and distribution of microorganisms within the granules is complex. Until now, changes in granular composition and activity can only be observed in granules collected from laboratories or full-scale reactors (Barr et al. 2010; Li et al. 2020; Zhang et al. 2019; Wilén et al. 2018). In general, SBRs are not the ideal choice for multivariate fundamental analyses due to their large volume, which hampers parallelized experiments with a high throughput. However, structured and fundamental microbiological and molecular biological investigations would be extremely helpful for further process understanding and optimization. Hence, a small-scale model system which enables multi parallel cultivation and screening under precise user-defined conditions would be desirable.

Recently, we introduced a microfluidic platform that enables the long-term cultivation of biofilms with simultaneous analysis and sampling (Hansen et al. 2019). In this system, polydimethylsiloxane (PDMS) chips are connected to automated liquid handling with analysis instrumentation. Furthermore, the system is operable under oxic and anoxic conditions and offers a high spatiotemporal resolution in the analysis of metabolites and biofilm composition making it an ideal platform for the development of biofilm-based model systems. In this study, we take advantage of this infrastructure with the aim to engineer a tailored system that can mimic the aerobic granular sludge process and enrich for PAOs. Molecular analysis of the inoculum and the developing biofilm microbiome in the microfluidic chips in combination with fluorescence microscopy analysis provides evidence for the successful enrichment of PAOs from activated sludge flocs.

## Materials and methods

### Setup of flow cell system

**Fig. 1:**
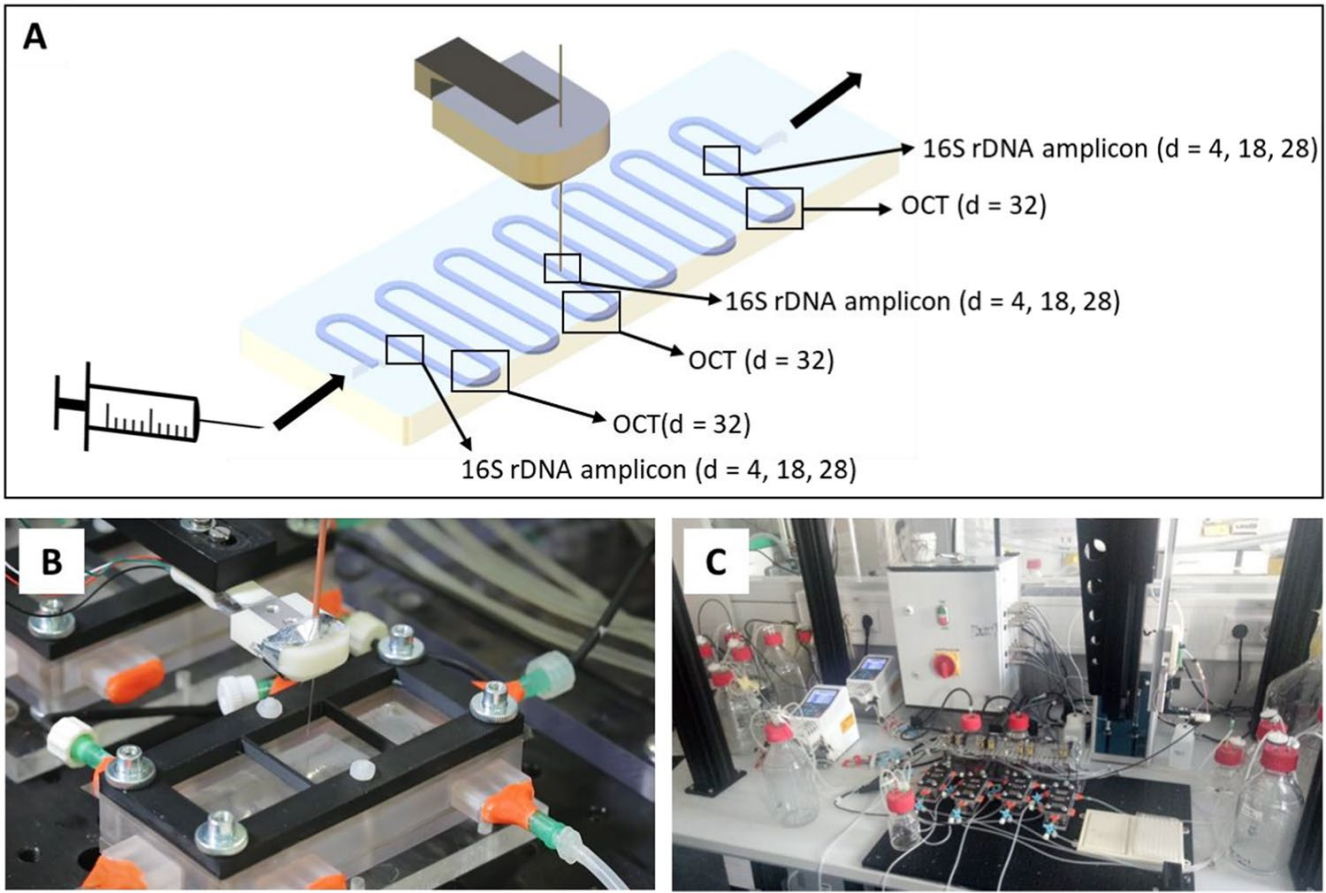
Anoxic cultivation of biofilms using microfluidic chips with simultaneous sampling and optical analysis. **A** The different sampling locations and time points are shown schematically. **B** Photo of a chip during sampling using the robotic device. The cannula is about to pierce through the plastic film to take a sample. **C** Photo of the whole infrastructure. On the left, the medium bottles and the peristaltic pumps are visible. In the center, the four cultivation chips can be seen. Behind them, the control box is located, which contains the programmable logic module. After leaving the chips, the medium flows into bottles, which are placed on the right side of the photo

The polydimethylsiloxane (PDMS) microfluidic chips with a meandering channel design were produced as recently described (Hansen et al. 2019). The microfluidic chip design was based on the dimension of standard microscope slides (76 × 26 mm^2^, DIN ISO 8037–1:2003–05) and the channel had a rectangular cross-section of 500 × 1000 μm^2^ and a total volume of 150 μL. Anaerobic cultivation conditions could be realized by the use of polycarbonate chambers, which can be constantly purged with nitrogen gas to enable anoxic conditions. The use of a plastic foil (0.043-mm thickness; Goodfellow GmbH, Hamburg, Germany) instead of hard plastic lids allowed withdrawing samples during ongoing anoxic cultivation directly from the cultivation channel using a robot-assisted sampling device (Fig. 1). Standard cannulas, BD Connecta™ micro-valves (Becton Dickinson, Franklin Lakes, New Jersey, USA), and luer lock fittings were used for connection to the medium supply. Media were supplied to the chips via silicone tubings (Carl Roth, Karlsruhe, Germany) for oxic media or via fluororubber tubings (Cole-Parmer, Vernon Hills, IL, US) for anoxic media. All tubes had an inner diameter of 1.5 mm. Constant medium supply was assured using peristaltic pumps (Reglo ICC, Cole-Parmer, Vernon Hills, IL, USA). Chips were operated in duplicates with flow rates of 2 mL h^−1^ and 4 mL h^−1^, respectively. Anoxic media bottles were constantly purged with N_2_ gas and oxic media bottles with compressed air. For the implementation of the alternating cycles of two different media, each chip was equipped with two 3/2 solenoid pinch valves (S305, Asco Numatics Sirai, Bussero, Italy). The supply of N_2_ gas and compressed air was achieved by means of two 2/2 solenoid valves (L172, Asco Numatics Sirai, Bussero, Italy). All solenoid valves were controlled via a programmable logic module (Logo! 8.2, Siemens, Munich, Germany).

### Inoculation and growth conditions

**Fig. 2:**
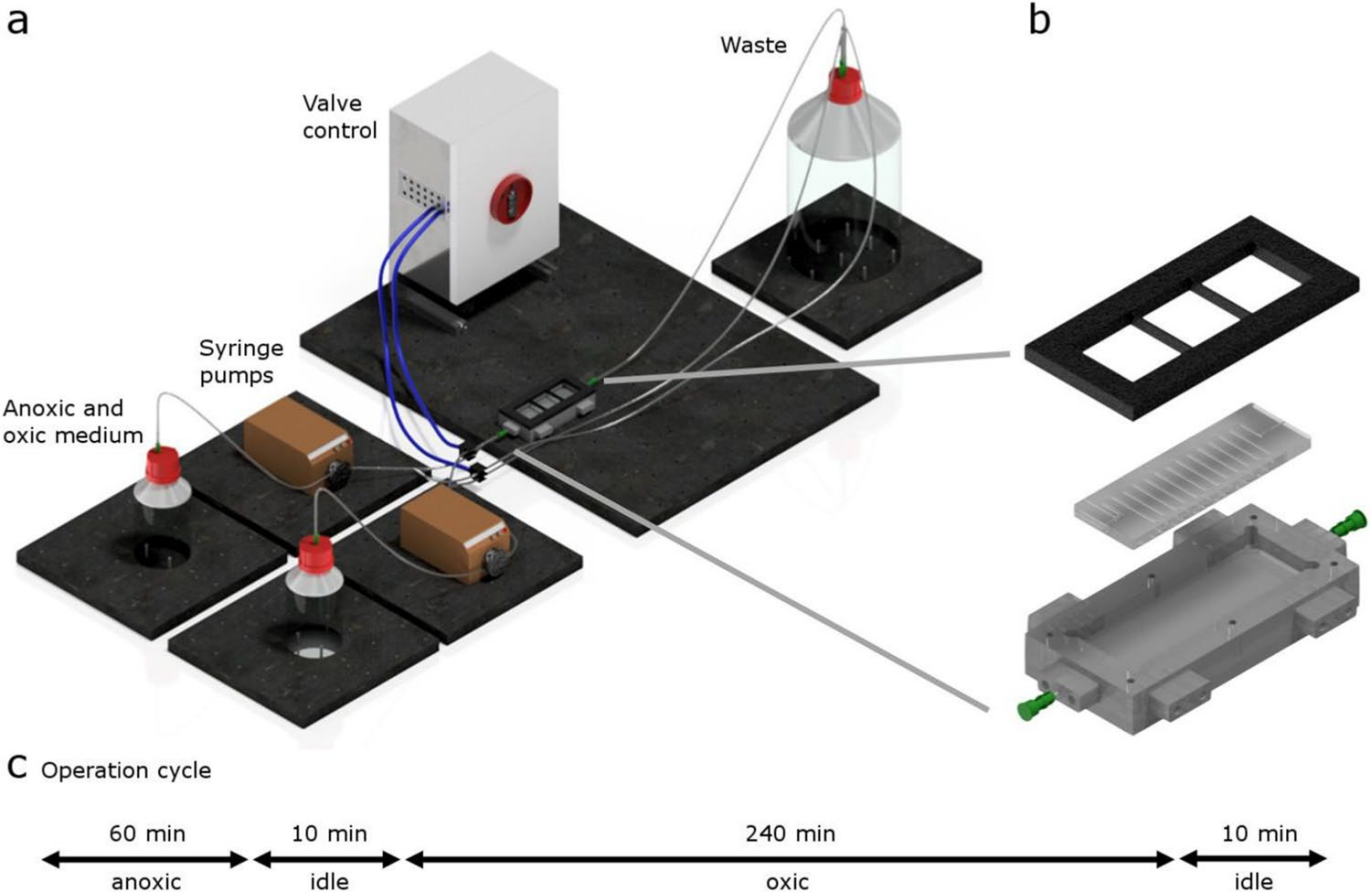
Setup of the experiment with one exemplary chip (**a**) and a detailed view of a chip in an anoxic chamber with a meandric channel (**b**). The medium is pumped from storage flasks via peristaltic pumps through the cultivation channel or directly into the waste storage depending on which phase is active. The flow is controlled by a programmable logic module via solenoid valves. The operation cycle is schematically shown in (**c**)

Activated sludge from the municipal sewage treatment plant Weinheim (Altau 10, 69469 Weinheim, Germany) was taken as inoculum. The treatment plant does apply biological phosphorus removal so it is reasonable that the sludge contains PAOs. The settled sample was decanted and thus reduced to about 30% of the original volume. The activated sludge was diluted 1:10 (v:v) with sterile ultrapure water and filtered using a 70-μm filter (Thermo Fisher Scientific, Waltham, Massachusetts, USA) which removed larger particles, leaving only planktonic cells and smaller biofilm flocs, which is crucial for the inoculation process in a microfluidic system. Prior to inoculation, the entire system was equilibrated with medium for 12 h. The inoculation was carried out with a Nexus3000 syringe pump (Chemyx, Stafford, USA) for 22 h at a flow rate of 0.6 mL h^−1^ via a separate side port of the PDMS chips (Fig. S1). At the same time, cultivation medium was supplied via the front port with 2 or 4 mL h^−1^, respectively. While inoculation was taking place, the logic module was already active, so that the phases described in the following were already alternating:

During a 1-h anoxic phase, the chips were supplied with anoxic carbon-containing medium (2.86 μM NH_4_Cl, 0.21 μM Na_2_HPO_4_ × 2 H_2_O, 0.11 μM KH_2_PO_4_, 8.33 mM C_2_H_3_NaO_2_ and 1 mL of Wolfe’s mineral elixir according to DSMZ recipe #792) and flushed with N_2_ gas. Between the main phases, short transition phases were implemented to prohibit a contamination of organic carbon-containing medium into the oxic phase and to establish anoxic conditions before switching to the feast medium. The transition phase was 10 min long, during which chips were supplied with oxic medium (2.86 μM NH_4_Cl, 0.21 μM Na_2_HPO_4_ × 2 H_2_O, 0.11 μM KH_2_PO_4_ and Wolfe’s mineral elixir as described above) under an anoxic atmosphere (N_2_). In the subsequent 4-h oxic phase, an oxic medium was supplied under aeration with compressed air. After another ten-minute transition phase, the cycle started again from the beginning (compare Fig. 2). Figure S1 shows a schematic diagram of the medium supply and gas flow during the anoxic and oxic phases. Cultivation took place at room temperature for 33 days.

### Optical coherence tomography (OCT)

Mesoscopic structures of biofilms were monitored using optical coherence tomography (OCT). OCT images were captured at three different positions (see Fig. 1) using a GANYMEDE-II spectral domain system (Thorlabs GmbH, Dachau, Germany) with an LSM04 objective lens. Parameters were chosen as described (Bauer et al. 2019) and datasets were processed with ImageJ/FIJI version 1.51 (Schindelin et al. 2012). To describe and quantify the biofilms, the acquired data sets were processed in two ways to compare the influence of the applied flow rates. First, biofilm volume was calculated by correlating the number of pixel signals with the voxel size of the images. Second, maps of three sections of the meandric channels were generated based on the process routine developed by Wagner and Horn (2017) to visualize the topography and local thickness of the biofilms.

### 16S rRNA gene amplification

Samples from each chip were taken from meandric sections (Fig. 1) via the robotic sampling device after 4, 18, and 28 days of cultivation. For 16S-sequencing analysis, the V3 region of the rDNA was amplified using the primers Illumina_ *Bakt_341F* (5′-ACA CTC TTT CCC TAC ACG ACG CTC TTC CGA *TCT CCT ACG GGN GGC WGC AG*-3′; Herlemann et al. 2011) and Illumina_*518R* (5′-GAC TGG AGT TCA GAC GTG TGC TCT TCC GAT CTW *TTA CCG CRG CTG CTG G*-3′; Lee et al. 2010). Each reaction contained 1 μL of the robotically withdrawn sample, 25 μL Phusion® Master Mix (New England Biolabs, Ipswich, Massachusetts, USA) and 2 μM forward and reverse primer, respectively. The cycling program involved two separate phases: a touchdown phase and a generic amplification stage. Initial denaturation was done for 5 min at 95 °C. The touchdown conditions were 95 °C for 40 s followed by an initial annealing temperature of 56 °C that was held for 40 s before the temperature was raised to 72 °C for 40 s. Subsequently, the annealing temperature was reduced by 1 °C each cycle, followed by an additional 23 cycles at the final annealing temperature of 51 °C and a final extension at 72 °C for 10 min. Each PCR reaction was performed in triplicate and subsequently pooled. The amplified DNA fragments were visualized on 2% agarose gels stained with MidoriGreen (Nippon Genetics, Tokyo, Japan) and the absence of contamination was checked by template-free negative controls. PCR products were gel purified using the Wizard® SV Gel and PCR Clean-Up System by Promega (Mannheim, Germany).

## SDNA extraction and library preparation for shotgun metagenome and amplicon sequencing

DNA from the filtered inoculum was extracted using the Dneasy® PowerBiofilm® Kit (Qiagen, Hilden, Germany) according to the manufacturer’s instructions. Extracted genomic DNA and amplified 16S rDNA were quantified using the Qubit dsDNA HS Assay Kit (Thermo Fisher Scientific, OR, USA). Libraries for shotgun and amplicon sequencing were prepared using the NEBNext® Ultra™ II FS DNA Library Prep Kit (New England BioLabs, Frankfurt, Germany), following the manufacturer’s instructions. The quality of the DNA libraries was verified using the Agilent High Sensitivity DNA Kit on the Agilent 2100 Bioanalyzer instrument (Agilent Technologies, Germany). The libraries were then sequenced on Illumina system 550 using a paired-end approach (NextSeq 500/550 High Output Kit v2.5, 150 Cycles).

## Bioinformatic analysis

Bioinformatic analysis of the 16S rRNA gene amplicon sequencing (merging, quality, and length trimming) was carried out with the CLC Genomic Workbench software 20.0.4 equipped with the additional microbial genomic module (Qiagen, Hilden, Germany). OTU clustering and phylogenetic analysis were performed with SILVA 16S v128 97% as the reference database. Since our amplicon samples showed an unequal sampling depth, we investigated the alpha diversity of the microbial communities of these samples which revealed that sufficient sequencing data was obtained to adequately describe the diversity of the microbial community (Fig. S2). Subsequently, the data sets were analyzed at the family level. Three data sets were obtained for each chip, from the front, middle, and back part of the cultivation channels. The read counts for each family for the three data sets were summed up to examine the mean abundance of each family over the entire cultivation channel. Due to technical difficulties, sequencing was not performed on day 28 for 4 mL h^−1^ (replicate 2) and for the front part of the chip 4 mL h^−1^ (replicate 1). Statistical significance was calculated using a one-sided one-sample *t*-test. Analysis of the metagenomic sequencing was performed using the SqueezeMeta pipeline version 1.2.0 in sequential mode (Tamames and Puente-Sánchez 2019). In summary, trimming and quality filtering were conducted using Trimmomatic (Bolger et al. 2014); MEGAHIT (Li et al. 2015) was used for assembly; removal of short contigs (< 200 bp) and contig statistics were done using PRINSEQ (Schmieder and Edwards 2011); RNAs were predicted using Barrnap (Seemann 2014); 16S rRNA sequences were taxonomically classified using the RDP classifier (Wang et al. 2007); tRNA/tmRNA sequences were predicted using ARAGORN (Laslett and Canback 2004); Prodigal (Hyatt et al. 2010) was employed for ORF prediction; similarity searches for GenBank (Clark et al. 2016), eggNOG (Huerta-Cepas et al. 2016), and KEGG (Kanehisa and Goto 2000) were performed using DIAMOND (Buchfink et al. 2014); HMM homology searches were conducted with HMMER3 (Eddy 2009) for the Pfam database (Finn et al. 2014) and Bowtie2 (Langmead and Salzberg 2012) was employed for read mapping against contigs. The mapping statistics of the metagenome can be seen in figure S3. File 11.mcount was used to obtain the phylogenetic composition of the metagenome. Furthermore, the presence of four different genes, namely *ppk*1, *ppk*2, *pha*C, and *pha*Z, was investigated by searching for the corresponding KEGG IDs (K00937, K22468, K03821, and K05973, respectively) within 13.orftable (Fig. S4). All raw sequencing data can be accessed via SRA accession SUB10937244.

## Fluorescence in situ hybridization (FISH) and confocal imaging

Since FISH protocols are based on the fixation of cultured cells, this method was performed as an endpoint analysis after 33 days of cultivation and with a sample from the inoculum. A fully automated FISH procedure was applied as recently described (Hansen et al. 2019) with probes PAO846 (Crocetti et al. 2000; 5′-GTT AGC TAC GGC ACT AAA AGG-3′ conjugated with FITC) and Gam42a (Crocetti et al. 2002; 5′-GCC TTC CCA CAT CGT TT-3′ conjugated with Cy5, and addition of Gam42a-Competitor (5′-GCC TTC CCA CTT CGT TT-3′)). All chips were counterstained with 4′,6-diamidino-2-phenylindole (DAPI). In addition, the fluorescent phenoxazine dye Nile Red (200 mg mL^−1^ in dimethyl sulfoxide) was used to stain hydrophobic cell components. Microscopic imaging of the flow cell was conducted on a confocal Leica TCS SP5 microscope and LASAF Version 2.6 software with a 63 × /1.40 objective (HCX PL APO).

## Results

### Advancing the microfluidic biofilm cultivation platform for alternating growth condition intervals

To establish a model reactor system for microbial communities in AGS, we extended our recently published microfluidic cultivation platform (Hansen et al. 2019). To this end, alternating oxic starvation and anoxic feeding phases were established by integration of a logic module, with controlled solenoid valves (Fig. 2). Between the longer phases for aerobic and anaerobic growth we established short transition phases to prohibit contamination of organic carbon-containing medium into the oxic phase and to establish anoxic conditions before switching to the feast medium. To investigate the responsiveness of the system and to establish a time frame for these transition phases, we conducted preliminary tests with two different dye solutions (Fig. S5). These experiments revealed that a duration of 10 min for the intermediate phases in the following experiments was sufficient. The microfluidic chips were embedded in a chamber to which a gas overpressure can be applied. To test for anoxic conditions in these chambers, we conducted an experiment with a biofilm of a *Shewanella oneidensis* strain expressing a red fluorescent protein. This protein needs oxygen in order to fold properly and gain its fluorescent properties (Heim et al. 1994). Hence, we grew the *S. oneidensis* biofilm first under fumarate reducing anoxic conditions and microscopically inspected after 3 days whether we would see fluorescent signals. As this was not the case, we switched to the oxic mode by replacing nitrogen with air overpressure and imaged the biofilm again. As is depicted in figure S6, fluorescently labeled cells could be detected within 60 min after the switch to oxic conditions. This result indicated that the system was capable of sufficiently lowering the oxygen concentration under at least 0.1 ppm of dissolved oxygen to prevent proper maturation of the RFP protein (Hansen et al. 2001).

The microfluidic platform was also advanced so that samples could be retrieved from the biofilm under oxic and anoxic conditions using the automatic biofilm sampler described previously (Hansen et al. 2019). To this end, the microfluidic chips were placed in the abovementioned chamber covered by a plastic film instead of hard plastic lids. Hence, samples could be taken directly from the cultivation channel even during anaerobic cultivation periods as the sample needle could simply puncture the plastic film cover. The constant overpressure in the gasket ensured stable oxic and anoxic conditions throughout the experiment.

### Establishing the quantity of PAOs in the activated sludge inoculum

**Fig. 3:**
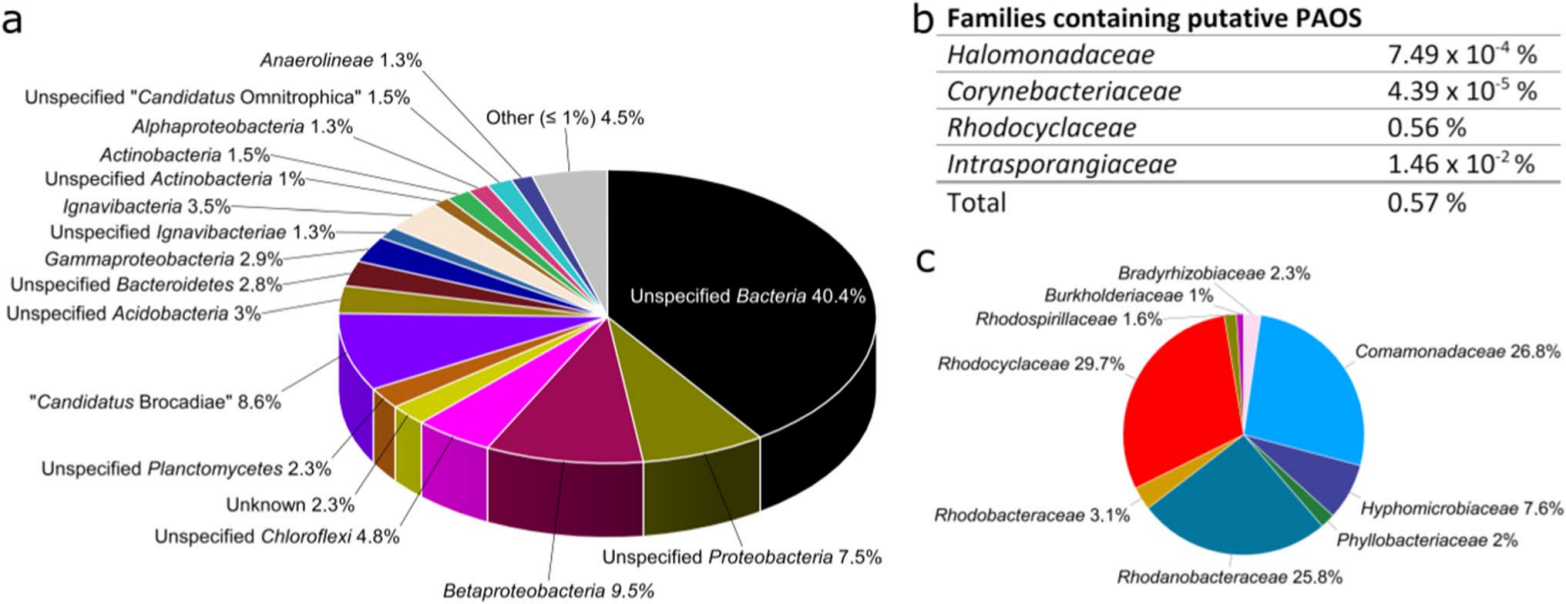
Phylogenetic evaluation of the inoculum. **a** The phylogenetic composition of the inoculum is shown as relative abundance at class level, based on shotgun metagenomic sequencing. **b** The fraction of putative polyphosphate-accumulating organisms (PAOs) in the inoculum according to the Microbial Database for Activated Sludge (MiDAS; Nierychlo et al. 2020). **c** Percentage distribution of families within the metagenome that have both *phaC*, *phaZ*, and at least one *ppk* gene version (*ppk1* or *ppk2*). Only taxa that could be identified to family level have been included

As the goal of our study was to establish a platform for the cultivation of PAO, we inspected whether a sample from an aeration tank of a municipal sewage plant would contain the desired organisms. To this end, we conducted a metagenomic study, to (I) analyze the community composition and (II) identify potential PAOs by inspecting the metagenome for key genes involved in polyphosphate and PHA synthesis and assign them to a phylogenetic group. As expected, the metagenomic analysis revealed a rather complex microbial community (Fig. 3a), including key organisms for ammonium oxidation (nitrification and anammox) and methanogenesis as typical aerobic and anaerobic wastewater sludge community members.

Within the assembled metagenome, we specifically searched for organisms belonging to the families *Intrasporangiaceae*, *Rhodocyclaceae*, *Obscuribacteriaceae*, *Corynebacteriaceae*, and *Halomonadaceae*. A number of organisms belonging to these families were proven to accumulate phosphate and are listed as such in the Microbial Database for Activated Sludge (MiDAS; Nierychlo et al. 2020). We could assign contigs to all of the above-listed families except the *Obscuribacteriaceae* (Fig. 3b). As the phylogeny does not necessarily have to reflect a metabolic trait, we also conducted a reverse analysis and searched for families that contain genes putatively encoding a polyphosphate kinase (*ppk1* or *ppk2*), a polyhydroxyalkanoate polymerase (*phaC*), and a polyhydroxyalkanoate depolymerase (*phaZ*). The analysis revealed nine families fulfilling these requirements of which the *Rhodocyclaceae* contributed to roughly one-third of the “TPMs” for the four genes (Fig. 3c). Of note, the term “TPM” was introduced by Tamames and Puente-Sánchez (2019). It is used for the statistical analysis of metagenome data analogous to the often-used TPM-based analysis of metatranscriptomes. As such, it gives a normalized abundance of reads for specific genes within a metagenomic dataset. Hence, the bioinformatic analysis allowed us with sufficient certainty to assume that PAOs were present in the inoculum and that a microfluidic enrichment campaign could be successful. Still, these families were highly underrepresented. Even the most abundant family *Rhodocyclaceae* contributed to less than 1% of the metagenomic reads.

**Fig. 4:**
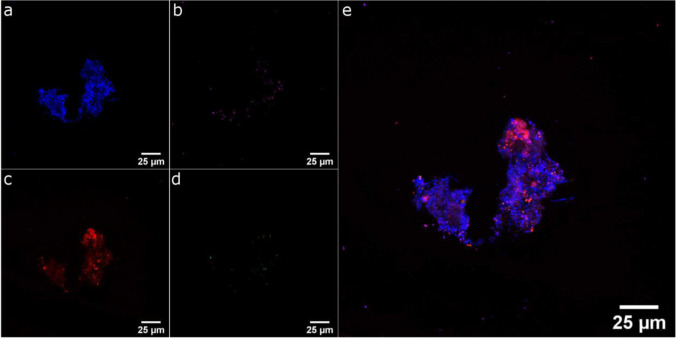
Fluorescence microscopic image of the inoculum using PAO-specific probe PAO846 (Crocetti et al. 2000; green; **d**), γ-proteobacteria-specific Gam42a-probe (Crocetti et al. 2002; magenta; **b**) and 4′,6-diamidino-2-phenylindole (DAPI; blue; **a**) as counterstain. The fluorescent phenoxazine dye Nile Red was used to stain hydrophobic cell components (red; **c**). The merged image is shown in **e**

We also aimed to corroborate the bioinformatic results with a FISH analysis of the biofilm flocs. The PAO-specific probe PAO846 was used which targets organisms belonging to the *Rhodocyclaceae* (Crocetti et al. 2000). Moreover, we stained the biofilm with the γ-proteobacteria probe Gam42a (Crocetti et al. 2002), as this would target among others glycogen-accumulating organisms (Günther et al. 2009). Last but not least, we used Nile Red, which binds to hydrophobic structures and is often used to stain PHA globules within the cells (Manz et al. 1992). As is depicted in Fig. 4, this analysis revealed a small fraction of cells that seemed to be positive for PAO846 and Nile Red. These results supported the metagenomic analyses and the deduced low abundance of PAOs in the inoculum.

### The mesoscopic structure of the biofilms after 32 days of cultivation suggests no impact of the investigated flow rates on biofilm characteristics

**Fig. 5:**
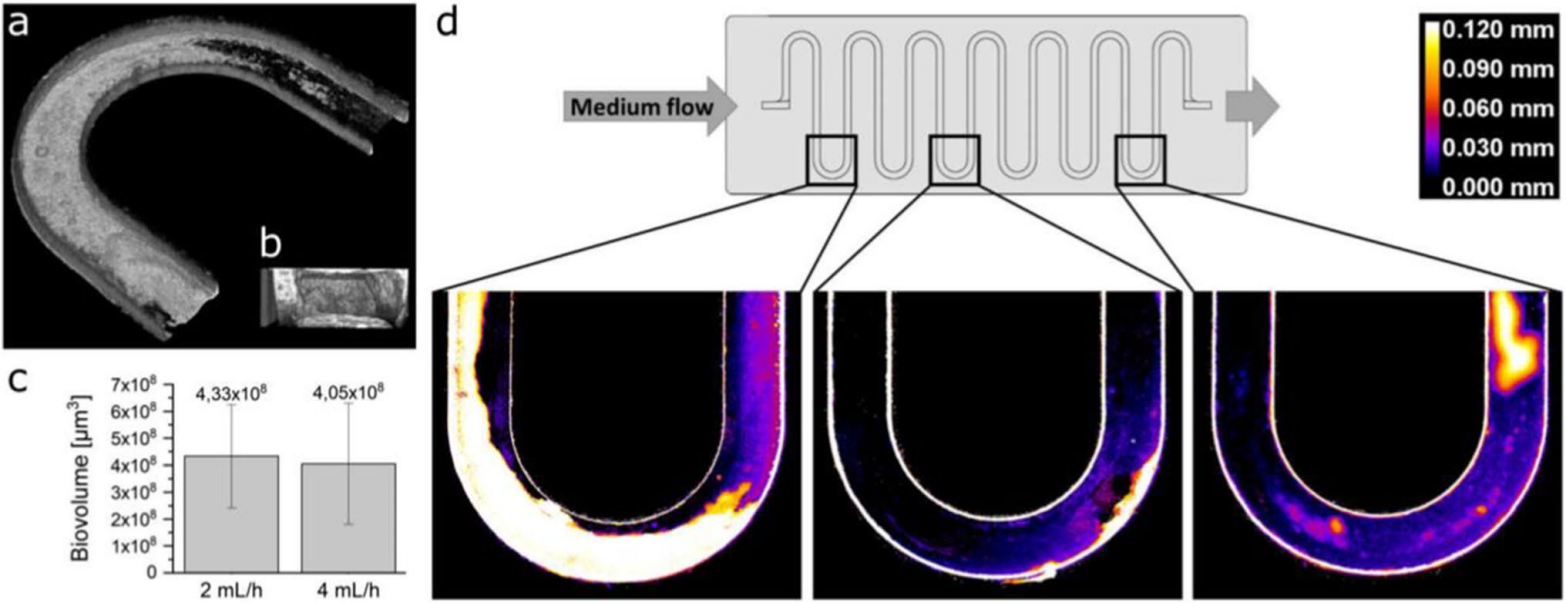
Investigation of the mesoscopic biofilm structures using optical coherence tomography (OCT). Representative visualizations of OCT measurements are shown in **a** as well as in **b**, the former showing an entire meander and the latter a cross-section of a channel. **c** Average biofilm volumes are shown as mean values with standard deviations for the two investigated flow rates. **d** Representative maps of three sections of meandric channels to visualize the topography and local thickness of biofilms are shown. The representative images derive from 4 mL/h (replicate 1)

The microfluidic reactors were inoculated using samples from the same batch of the municipal sewage plant as described above. After 32 days of incubation, an optical coherence tomography analysis was conducted in order to assess whether the mesoscopic structure of the biofilms under the two flow regimes would deviate from each other (Fig. 5). We hypothesized that the substrate concentrations in the medium should not limit biofilm growth as the substrates were added in excess regarding the available surface in the microfluidic reactors. Hence, only the difference in the shear forces that were applied to the duplicate reactors could have affected the developing biovolume. Still, the two flow rates 2 mL/h (1.1 mm/s) and 4 mL/h (2.2 mm/s) led to comparable average biofilm volumes of 4.05 and 4.33 × 10^8^ μm^3^, respectively and the difference of the biovolume was not statistically significant at either of the three observed measurement points (Fig. 5; Fig. S7). Also, the average biofilm height (RC) of 31.5 and 33.7 μm was comparable for the two investigated flow rates. The difference in shear forces was not large enough to generate different biofilm morphologies. Moreover, the biofilm growth was apparently not limited by the substrate concentration or by mass transfer limitations. To this end, at least 50% of the acetate that was added with the medium under anoxic conditions could be detected at the outflow of the chip throughout the entire timeframe of the experiment (data not shown).

### Amplicon-based sequencing and FISH show enrichment of *Rhodocyclaceae*

Biofilm samples were extracted via the sampling robot after 4, 18, and 28 days of continuous operation and used as a template for amplicon sequencing. Of note, the obtainable sample size was limited due to the flow rate through the microfluidic chip, the biofilm thickness, and the time frame of the individual oxic and anoxic phases. The small sample volume together with the low biofilm thickness precluded the use of an amplification-free sequencing approach. Due to the defined medium composition and process parameters compared to the wastewater treatment plant, we observed an expected rather drastic change of the community composition over the time course of the experiment. We could not observe clear flow rate-dependent differences between the two investigated duplicates (2 mL h^−1^ vs. 4 mL h^−1^), which might be expected as the flow rate had no significant impact on biofilm characteristics as detected via OCT analysis.

**Fig. 6:**
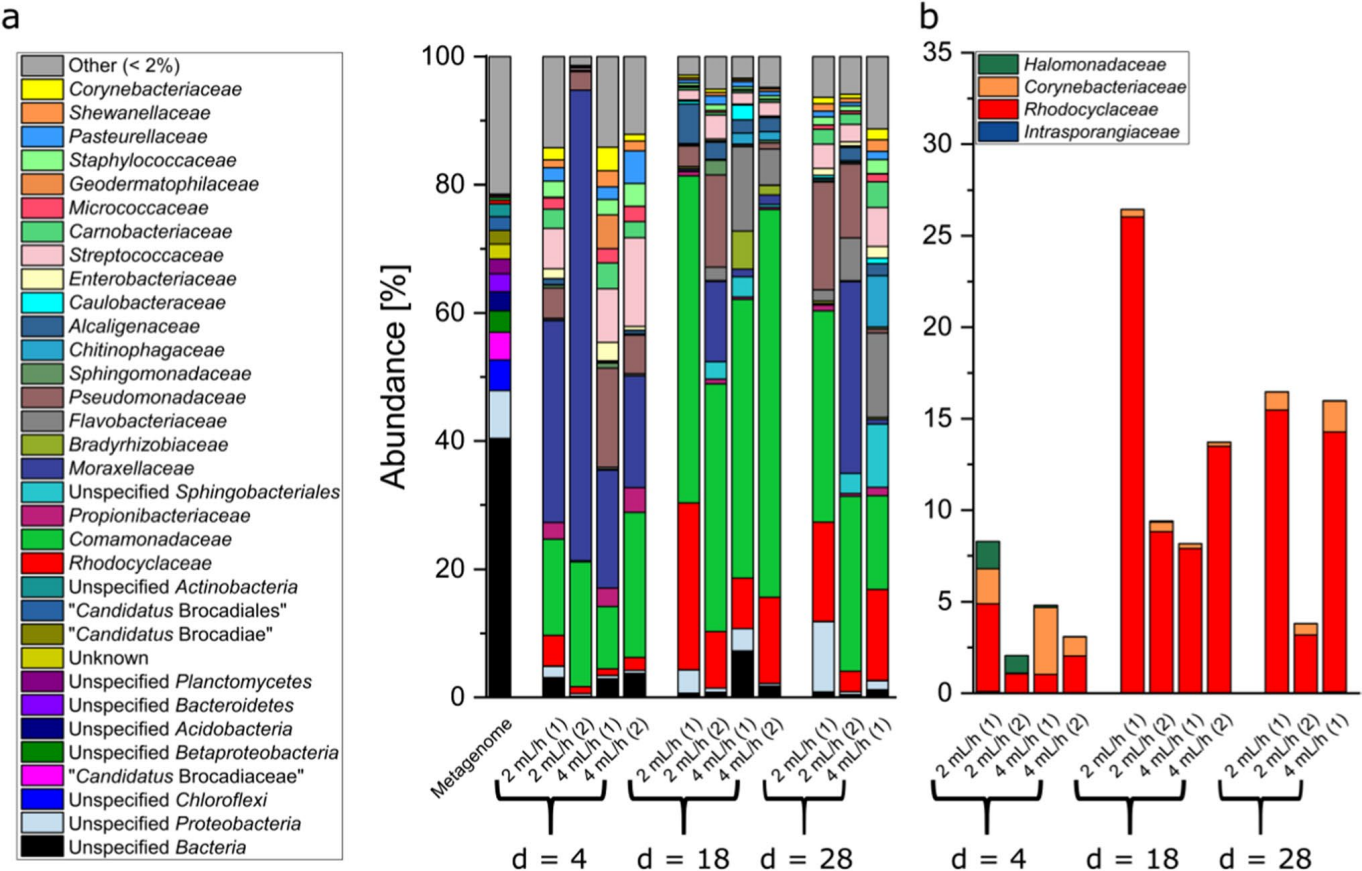
Phylogenetic evaluation of the matured biofilms after 4, 18, and 28 days using amplicon sequencing for the two flow velocities. The individual bars represent the mean read counts for each family at the three sampling points. This allowed us to examine the abundance of each family over the entire cultivation channel. **a** Phylogenetic composition of the metagenome and the biofilms is shown as the relative frequency at the family level. **b** The fraction of putative polyphosphate-accumulating organisms (PAOs). Due to technical errors, we were not able to retrieve an amplicon sequencing result from one of the chips after 28 days of operation

**Fig. 7:**
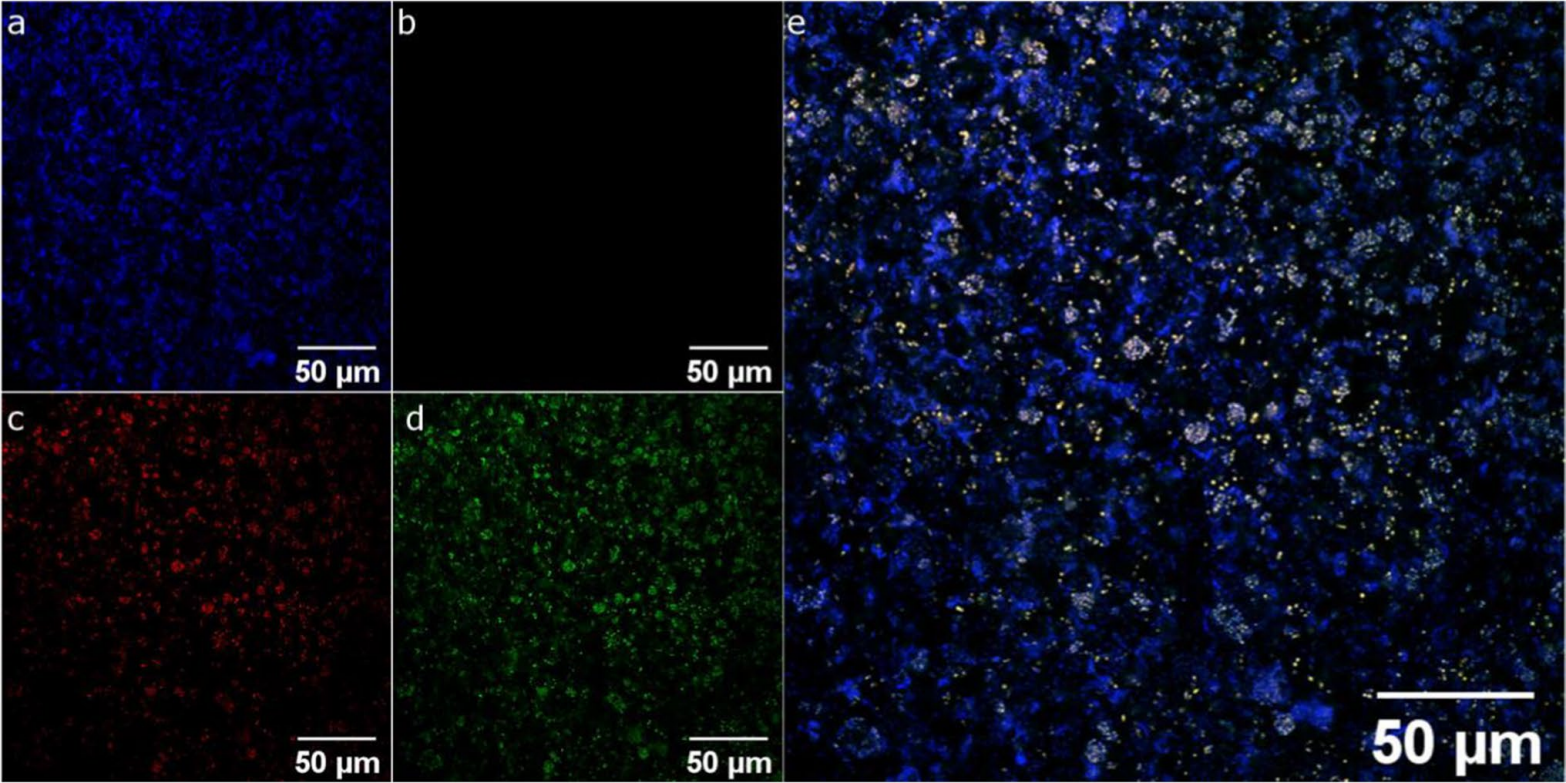
Representative fluorescence microscopic image of matured biofilm (4 mL h^−1^, replicate 1) within the microfluidic flow channel using PAO-specific probe PAO846 (Crocetti et al. 2000; green; **d**), γ -proteobacteria-specific Gam42a-probe (Crocetti et al. 2002; magenta; **b**) and 4′, 6-diamidino-2-phenylindole (DAPI; blue; **a**) as counterstain. The fluorescent phenoxazine dye Nile Red was used to stain hydrophobic cell components (red; **c**). The merged image is shown in **e**

We could observe a significant (*p*-value = 0.01) and on average 19-fold (± 11) enrichment of organisms belonging to the *Rhodocyclaceae* over the cultivation period, which provides clear evidence for the successful application of the feast famine cycles via the chip infrastructure (Fig. 6b). Other potential PAOs were more abundant at day four and decreased in abundance from day four to day 18. As the *Rhodocyclaceae* comprised a considerable proportion of the overall community at the end of the analysis, we aimed to bolster the phylogenetic evidence for PAO enrichment via FISH analysis. Since FISH protocols are based on the fixation of cultured cells, this method is only applicable as endpoint analysis. Generally, the abundance of *Rhodocyclaceae* increased when comparing the FISH images of the inoculum (Fig. 4) with images of the microfluidic chip biofilms at the end of the enrichment experiment (Fig. 7).

Besides the enrichment of putative PAOs, the remaining microbiome in the microfluidic chips developed with a dynamic community composition. Whereas members of the *Moraxellaceae* dominated at day four of cultivation they were mostly replaced by organisms belonging to the *Comamonadaceae* at day 18 (Fig. 6). Furthermore, we could not sustain the growth of members of the Planctomycetes with the chosen experimental settings. This suggests that the physiological ability of anaerobic ammonium oxidation was lost in the microfluidic culture at least to some extent.

## Discussion

In this manuscript, we introduce a model system for aerobic granular sludge on a small scale by using a machine-assisted microfluidic cultivation platform. With an implemented logic module controlling solenoid valves alternating oxic hunger and anoxic feeding phases could be realized (Fig. 2). Sampling during ongoing anoxic cultivation directly from the cultivation channel was achieved with a robotic sampling device (Fig. 1). Thus, the developed platform enables multi-parallel microfluidic cultivation under user-defined intervals of different growth conditions.

Since the aim of our study was to establish a platform for the cultivation of PAOs, we screened the potential inoculum for the desired organisms. Metagenomic analysis revealed a rather complex microbial community (Fig. 3a), of which 0.57% of the families could be assigned to putative PAOs (according to MiDAS; Nierychlo et al. 2020). Biofilm cultivation under alternating cycles of oxic starvation and anoxic feeding phases resulted in the desired enrichment of putative PAOs and, in particular, enrichment of species from the family *Rhodocyclaceae*. Although other potential PAOs could also be detected at the beginning of the cultivation, their abundance decreased during the experiment (Fig. 6). Hence, the developed technology can be applied to mimic aerobic granular sludge processes and to enrich for key species. In fact, the here described community development and enrichment of members of the *Rhodocyclaceae* can be compared to a study by Lv et al. (2014). The authors observed during the maturation process of aerobic granules in larger scale sequencing batch reactors an enrichment of similar microbial families including the *Rhodocyclaceae*. As the here described microfluidic reactors can be set up in a multi-parallel fashion, it will now be possible to automatically screen a wide variety of parameters and observe their impact on PAOs but also on other key species of the process. Other studies aiming at the isolation or enrichment of PAOs used SBRs with a volume of at least 2 L (Lu et al. 2006; Gao et al. 2019; Marques et al. 2018; Cokro et al. 2017). While this strategy led to the successful enrichment of PAOs, the reactor volume will hamper the time-efficient screening of the impact of process parameters on PAO quantity and activity. In fact, there is so far no robust guideline on how biological phosphate elimination can be supported by specific process variables (Nielsen et al. 2019). Only studies allowing multivariate and multi-parallel analyses will allow in the future to foresee the impact of individual process variables on a microbial process in a diverse process environment. Space and time limitations will not allow to conduct the necessary number of experiments in so far chosen SBRs.

Apart from the *Rhodocyclaceae*, the microbiome in the microfluidic chips developed with a dynamic community composition (Fig. 6). While members of the *Moraxellaceae* dominated at day four of cultivation, they were mostly replaced by organisms belonging to the *Comamonadaceae* at day 18. Interestingly, many members of both families have similar physiological abilities, as they are typically aerobic respiratory organisms. Hence, it is tempting to suggest that members of the two families might have been competitors for the same ecological function within the biofilm. Still, more than the *Moraxellaceae*, the *Comamonadaceae* include organisms with a wide range of physiological abilities and although many are aerobic heterotrophs this might not necessarily apply for the here enriched organisms. On day 28, members of the *Comamonadaceae* belonged still to one of the most abundant families but their abundance generally decreased. We could not sustain the growth of members of the Planctomycetes with the chosen experimental settings. We hypothesize that this might be due to the rather slow growth rates of the organisms and the rather thin biofilm, which will hamper the growth of anaerobic microorganisms because the respiratory oxygen consumption might be simply not sufficient to establish anaerobic niches within the oxic growth phases. Nevertheless, this process limitation could be tackled by reducing the concentration of dissolved oxygen in the oxic phases at least in the first days of the experiments and we will approach this issue in the following cultivation campaigns.

The system presented can mimic quite drastic changes like the abrupt shift from oxic to anoxic culture media described here, but they could in fact be steered into any direction, for instance, to direct the growth of facultative autotrophs. It was not only the aim to describe a method but to apply it for the enrichment of key species for aerobic granular sludge formation, which is difficult in laboratory cultures and large-scale aerobic granular reactors. The here provided data clearly revealed that it was possible to enrich for PAOs from the family *Rhodocyclaceae*. The amplicon sequencing results also display a rather uniform enrichment pattern and development of the microbiomes considering that we started with a highly diverse inoculum. The latter shows that the microfluidic system is able to generate reproducible results. At present, the amount of sample that could be retrieved from the chips did not allow for any chromatographic analyses. These would help to corroborate PAO activity by measuring phosphate concentrations. Moreover, sample size limitations did also not allow for metagenomic or transcriptomic sequencing using standard methods. This is why we relied in this study on amplicon sequencing, although it is clear that this method comes along with a typical PCR bias. In future studies, it will be possible to use genome or transcriptome amplification approaches to reach enough material for genomic and transcriptomic sequencing. Although the necessary multiple displacement amplification is also associated with sequencing bias, the risk of erroneous analysis is substantially reduced. Nevertheless, combining the robotic sampling with the recently established life cell FISH techniques could allow to target specific cells within the reactor, sort them using fluorescence assisted cell sorting or single-cell printing and aim for the cultivation of pure cultures or pure culture biofilms by specifically depositing the cells in sterile biofilm cultivation chips (Batani et al. 2019).

## Supplementary Information

Below is the link to the electronic supplementary material.

Supplementary file1 (PDF 4.00 MB)

## Data Availability

All raw sequencing data can be accessed via BioProject accession PRJNA687370. The data sets generated during and/or analyzed during the current study are either shown in the manuscript or available from the corresponding author on reasonable request.

## Code availability

The optical coherence tomography was used with the ThorImageOCT software version 5.2.1. Microscope software LASAF Version 2.6 was used. Optical coherence tomography datasets were processed with ImageJ/FIJI version 1.51.

Bioinformatic analysis of the 16S rRNA gene amplicon sequencing (merging, quality, length trimming, and alpha diversity analysis) was carried out with the CLC Genomic Workbench software 20.0.4 equipped with the additional microbial genomic module (Qiagen, Hilden, Germany). Analysis of the metagenomic sequencing was performed using the SqueezeMeta pipeline version 1.2.0.
