# Supplementary material for: Enrichment of phosphate-accumulating organisms (PAOs) in a microfluidic model biofilm system by mimicking a typical aerobic granular sludge feast/famine regime
Source: Appl Microbiol Biotechnol. 2022 Jan 15;106(3):1313–24. doi: 10.1007/s00253-022-11759-8 (PMC8816403; doi:10.1007/s00253-022-11759-8)
Supplement: Supplementary file 1 — Supplementary file1 (PDF 4.00 MB) [file 253_2022_11759_MOESM1_ESM.pdf]

SUPPLEMENTARY INFORMATION

Supporting and additional information of our findings. This file contains images and data which are meant to give a better idea of our experiment and findings as well as results of side and evaluating experiments.

Journal name: Applied Microbiology and Biotechnology

Article title: Enrichment of phosphate-accumulating organisms (PAOs) in a microfluidic model biofilm system by mimicking a typical aerobic granular sludge feast/famine regime

Edina Klein[1,2], Janek Weiler[1], Michael Wagner[2,3], Minja Čelikić[2], Christof M. Niemeyer[2], Harald Horn[3], Johannes Gescher[1,2]*

[1]Institute for Technical Microbiology (TMI), Hamburg University of Technology (TUHH), Kasernenstr. 12, 21073 Hamburg, Germany

[2]Institute for Biological Interfaces (IBG-1), Karlsruhe Institute of Technology (KIT), Herrmann-von-Helmholtz Platz 1, 76344 Eggenstein-Leopoldshafen, Germany

[3]Engler-Bunte-Institut (EBI), Water Chemistry and Water Technology, Karlsruhe Institute of Technology (KIT), Engler-Bunte-Ring 9a, 76131 Karlsruhe

*Correspondence and requests for materials should be addressed to J.G. (email: johannes.gescher@tuhh.de).

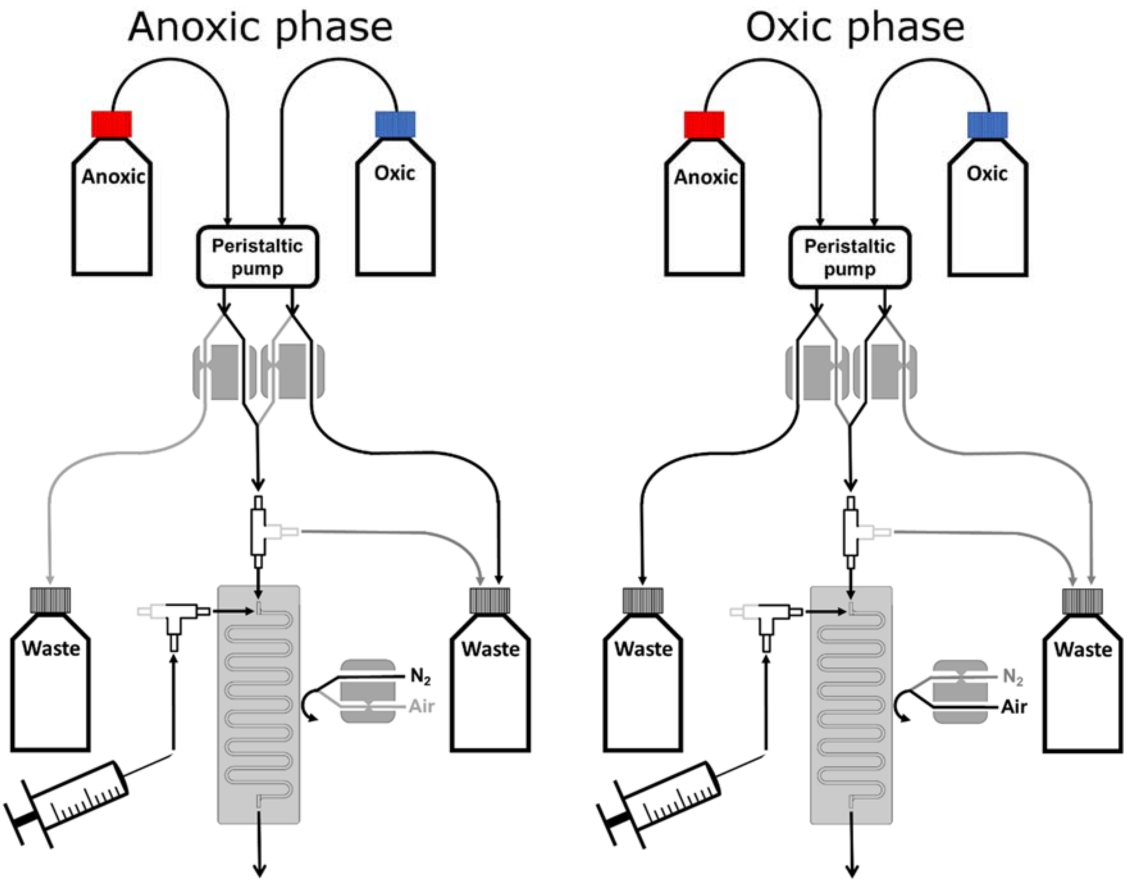

S1. Schematic diagram of the medium supply and gas flow during the anoxic and oxic phases. During the anoxic phase (left), the anaerobic carbon-containing medium is pumped into the microfluidic chips and the oxic medium into waste bottles. During the oxic phase (right), it is reversed. In addition, the separate side port used for inoculation (see syringe) is visible.

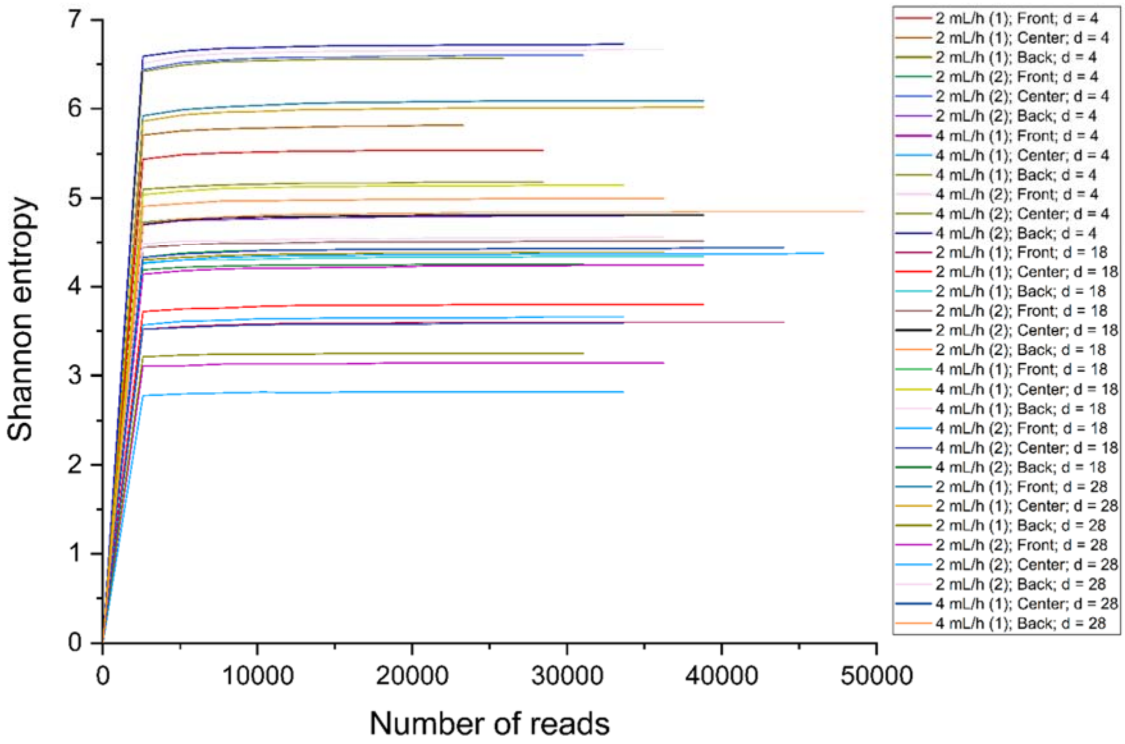

S2. Rarefaction curves of alpha diversity shown as Shannon entropy (Shannon 1997) of the microbial communities. Since our samples showed unequal sampling depth, we investigated alpha diversity of the microbial communities of the samples. Diversity analyses were carried out using CLC Genomic Workbench software 20.0.4 equipped with the additional microbial genomic module (Qiagen, Hilden, Germany).

A

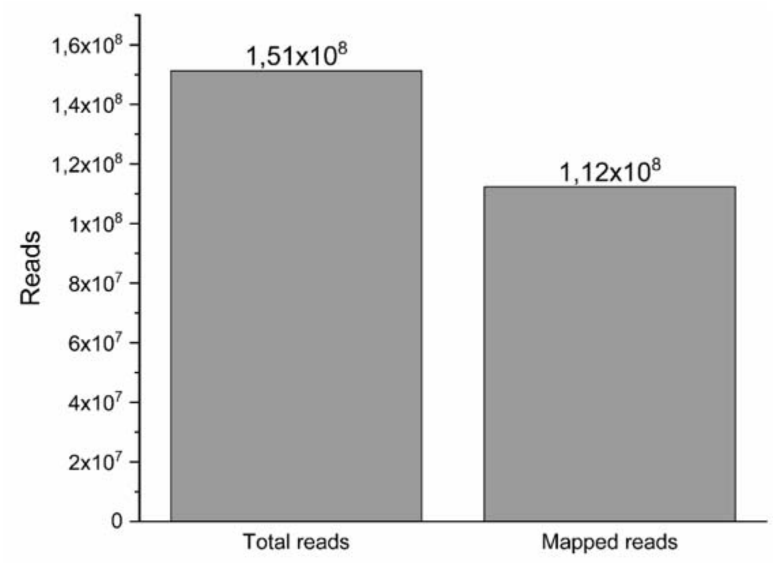

B

**Total reads**	151,380,100
**Mapped reads**	112,366,018
**Mapping perc**	74.23 %
**Total bases**	22,858,395,100

S3. Mapping statistics of the metagenome. **A)** Total and mapped reads are shown as well as **B)** relevant numbers for mapping statistics.

S4. Taxa within the inoculum that contain at least one of the following genes: *ppk1*, *ppk2*, *phaC*, *phaZ*. Note that the nomenclature transcripts per million (TPM) is not the correct term, as this is a metagenomic evaluation. Nevertheless, since this is an established term and to remain consistent with previous work, we follow the argumentation of Tamames and Puente-Sánchez and maintain the nomenclature "TPM" (Tamames and Puente-Sánchez 2019).

	**„TPM"**						
**Family**	***ppk1*** (K00937)	***ppk2*** (K22468)	***phaZ*** (K03821)	***phaC*** (K05973)	**Total**		
Bradyrhizobiaceae	0.557	0	0.482	0.479	1.518	Taxa having at least one *ppk* gene and *phaC* and *phaZ*	Families included in figure 3 c
Comamonadaceae	2.895	2.19	5.053	7.625	17.763		
Hyphomicrobiaceae	0.914	0.753	2.387	0.969	5.023		
Phyllobacteriaceae	0.454	0	0.422	0.429	1.305		
Rhodanobacteraceae	5.237	2.578	6.917	2.36	17.092		
Rhodobacteraceae	0.751	0.129	0.673	0.527	2.08		
Rhodocyclaceae	8.939	0	5.402	5.332	19.673		
Rhodospirillaceae	0.395	0	0.212	0.457	1.064		
Burkholderiaceae	0	0.101	0.368	0.175	0.644		
Unspecified *Alphaproteobacteria*	3.195	1.118	2.368	9.373	16.054		
Unspecified *Betaproteobacteria*	2.644	13.283	2.996	12.887	31.81		
Unspecified *Burkholderiales*	0.669	3.897	11.883	9.993	26.442		
Unspecified *Proteobacteria*	6.344	8.82	1.101	16.665	32.93		
Unspecified *Rhizobiales*	3.561	1.359	2.432	1.74	9.092		
Acetobacteraceae	0.397	0	0	0.158			
Alcanivoracaceae	0.369	0	0	0.184			
Bdellovibrionaceae	0.344	0	0	0			
Campylobacteraceae	0.136	0	0	0			
Caulobacteraceae	0.084	0	0.069	0			
Chitinophagaceae	0.059	0	0	0			
Dietziaceae	0.142	0	0	0			
Moraxellaceae	0.515	0.219	0	0.722			
Ectothiorhodospiraceae	0.078	0	0	0			
Intrasporangiaceae	2.057	0.32	0	0			
Flavobacteriaceae	0.225	0	0	0			
Gaiellaceae	0.254	0	0	0			
Mycobacteriaceae	0.12	0.791	0	0			
Ignavibacteriaceae	10.437	0	0	0			
Propionibacteriaceae	1.021	1.945	0	0			
Isosphaeraceae	0.097	0	0	0			
Unspecified *Actinobacteria*	7.251	7.656	0	3.644			

Methanosarcinaceae	0.278	0	0	0
Unspecified *Bacteria*	70.205	21.202	0	5.098
Methylocystaceae	0.208	0	0	0
Unspecified *Bacteroidetes*	7.859	1.864	0	2.866
Unspecified *Acidimicrobiia*	1.724	0.185	0	0
Unspecified *Acidobacteria*	20.408	2.054	0	0
Nitrosomonadaceae	11.756	0	0	0
Unspecified *Chloroflexi*	18.221	6.798	0	1.804
Unspecified *Anaerolineae*	0.527	2.114	0	0
Planctomycetaceae	0.563	0	0	0
Unspecified *Cyanobacteria*	1.526	0.683	0	0.113
Porphyromonadaceae	0.195	0	0	0
Unspecified *Dehalococcoidia*	0.219	1.881	0	0.104
Unspecified *Deltaproteobacteria*	0.09	0	0	5.292
Porticoccaceae	0.075	0	0	0
Unspecified *Gammaproteobacteria*	7.899	0.626	0	27.218
Unspecified *Bacteroidales*	0.838	0.237	0	0
Spirochaetaceae	0.394	0	0	0
„*Candidatus* Sumerlaeota“	0.078	0.057	0	0
Unspecified *Euryarchaeota*	0.214	0.086	0	0
Unspecified *Bdellovibrionales*	0.093	0	0.306	0
Unspecified *Caldilineae*	0.078	0	0	0
„*Candidatus* Omnitrophica“	4.87	0	0	0
Unspecified *Cellvibrionales*	0.159	0	0	0
Unspecified *Chlorobi*	0.572	0	0	0
Unspecified *Clostridiales*	0.439	0	0	0
Unspecified *Flavobacteriales*	0.052	0	0	0
Unspecified *Ignavibacteria*	0.132	0	0	0
Unspecified *Ignavibacteriae*	0.265	0	0	0
Unspecified *Ignavibacteriales*	0.681	0.373	0	0
Unspecified *Lentisphaerae*	0.375	0.265	0	0
Unspecified *Methanomassiliicoccales*	0.42	0.16	0	0
Unspecified *Methanomicrobiales*	0.889	0	0	0
Unspecified *Myxococcales*	0.88	0	0	0
Unspecified *Phycisphaerae*	0.3	0.137	0	0.141
Unspecified *Planctomycetales*	0.07	0	0	0

Unspecified *Planctomycetes*	2.283	0.327	0	0.42
Unspecified *Planctomycetia*	0.364	0	0	0.08
Unspecified *Propionibacteriales*	1.046	0.303	0	0
Unspecified *Rhodospirillales*	0.288	0	0	1.302
Unspecified *Solirubrobacterales*	0.322	0.322	0	0
Unspecified *Sphingobacteriales*	1.249	0	0	0
Unspecified *Sphingobacteriia*	0.183	0	0	0
Unspecified *Spirochaetales*	0.105	0	0	0
Unspecified *Spirochaetes*	0.061	0	0	0
Unspecified *Thermomicrobiales*	0.569	0	0	0
Unknown	2.82	1.458	0	0.378
Unspecified *Verrucomicrobia*	2.355	0.715	0	0.062
Unspecified *Verrucomicrobiales*	0.815	0	0	0
Unspecified *Xanthomonadales*	0.091	0	0	0.042
Xanthomonadaceae	0.172	0	0	0
Actinomycetaceae	0	0.124	0	0
Aeromonadaceae	0	0.086	0	0
Anaerolineaceae	0	0.105	0	0
Caldilineaceae	0	0.096	0	0
Frankiaceae	0	0.142	0	0
Gordoniaceae	0	0.527	0	0
Microbacteriaceae	0	0.12	0	0
Streptomycetaceae	0	0.066	0	0
Unspecified *Bacillales*	0	2.248	0	0
„*Candidatus* Dadabacteria“	0	0.128	0	0.144
„*Candidatus* Gracilibacteria“	0	0.068	0	0
„*Candidatus* Melainabacteria“	0	0.069	0	0
Unspecified *Corynebacteriales*	0	0.139	0	0.128
Unspecified *Firmicutes*	0	0.17	0	0
Unspecified *Nitrosomonadales*	0	0.077	0	0
Unspecified *Nitrospira*	0	0.282	0	0
Unspecified *Rhodocyclales*	0	0.279	0	5.3
Unspecified *Syntrophobacterales*	0	0.091	0	0
Methylobacteriaceae	0	0	0.133	0.089
Legionellaceae	0	0	0	0.763
Oxalobacteraceae	0	0	0	0.11

Rhodobiaceae	0	0	0	0.183
Sphingobacteriaceae	0	0	0	0.957
Sphingomonadaceae	0	0	0	0.333
Syntrophomonadaceae	0	0	0	0.385
„*Candidatus* Rokubacteria“	0	0	0	0.091
Unspecified *Clostridia*	0	0	0	0.03
Unspecified *Holophagae*	0	0	0	0.992
Unspecified *Oceanospirillales*	0	0	0	0.152
Unspecified *Sphingomonadales*	0	0	0	0.197
Bacteriovoracaceae	0	0	0.172	0
Rhizobiaceae	0	0	0.054	0
Unspecified *Oligoflexia*	0	0	0.113	0
Unspecified *Thermoleophilia*	0	0	0.115	0

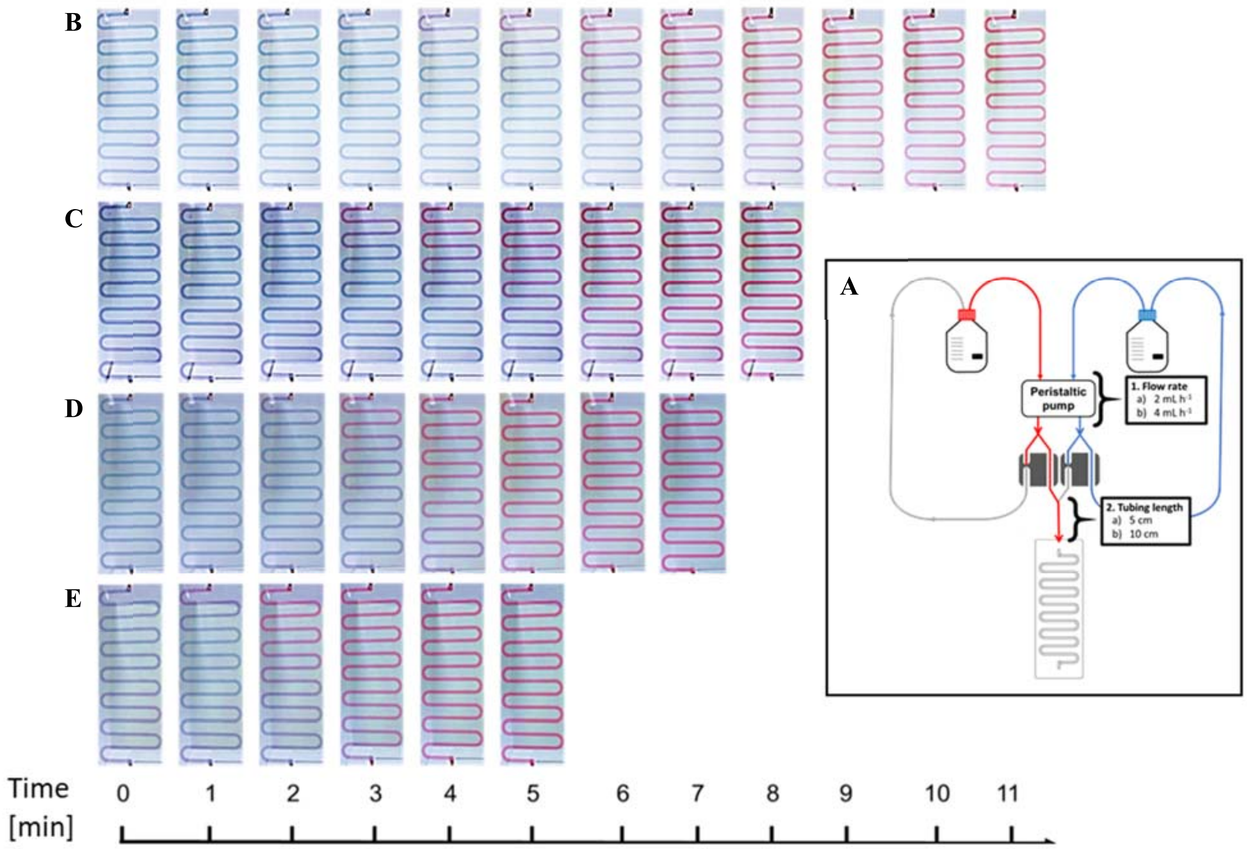

S5. Validation of the experimental setup for the cultivation in microfluidic chips, under cyclic change of two dye solutions. At time point t = 0 the solenoid valves (dark gray) were switched so that instead of blue fluid, red fluid was pumped into the chip. **A)** A scheme of the experimental setup is shown. Two flow rates (2 and 4 mL h^{-1}) with different distances of the valves to the chip (5 and 10 cm) were examined. **B)** 2 mL h^{-1}, 10 c m. **C)** 2 mL h^{-1}, 5 cm. **D)** 4 mL h^{-1}, 10 cm. **E)** 4 mL h^{-1}, 5 cm.

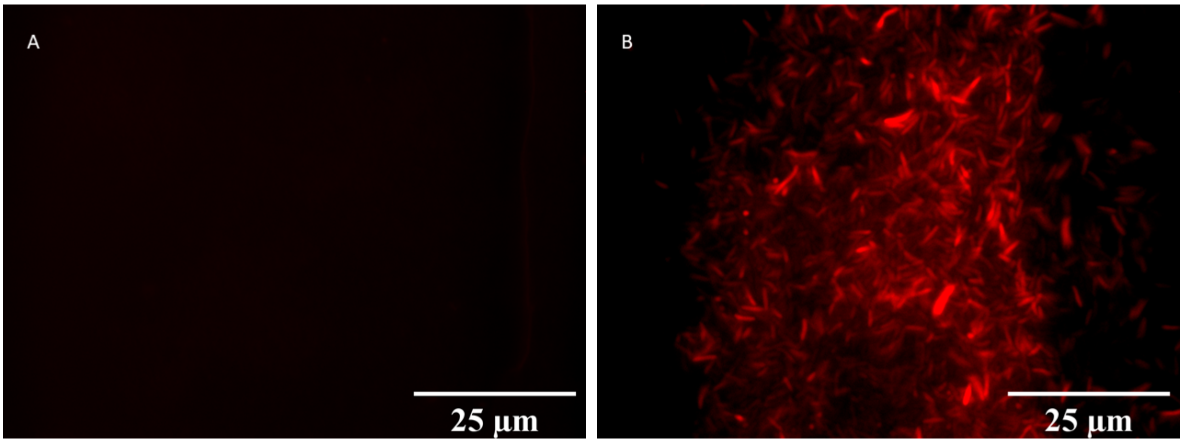

S6. Confirmation of anoxic conditions inside the cultivation channels. *Shewanella oneidensis* cells carrying a pMK-RQ plasmid with a red fluorescent protein (RFP) gene were grown for three days under anoxic conditions prior to the microscopic analysis **(A)**. Following, the nitrogen replenishment was disconnected, and images were taken again **(B)** revealing clear RFP signals under oxic conditions.

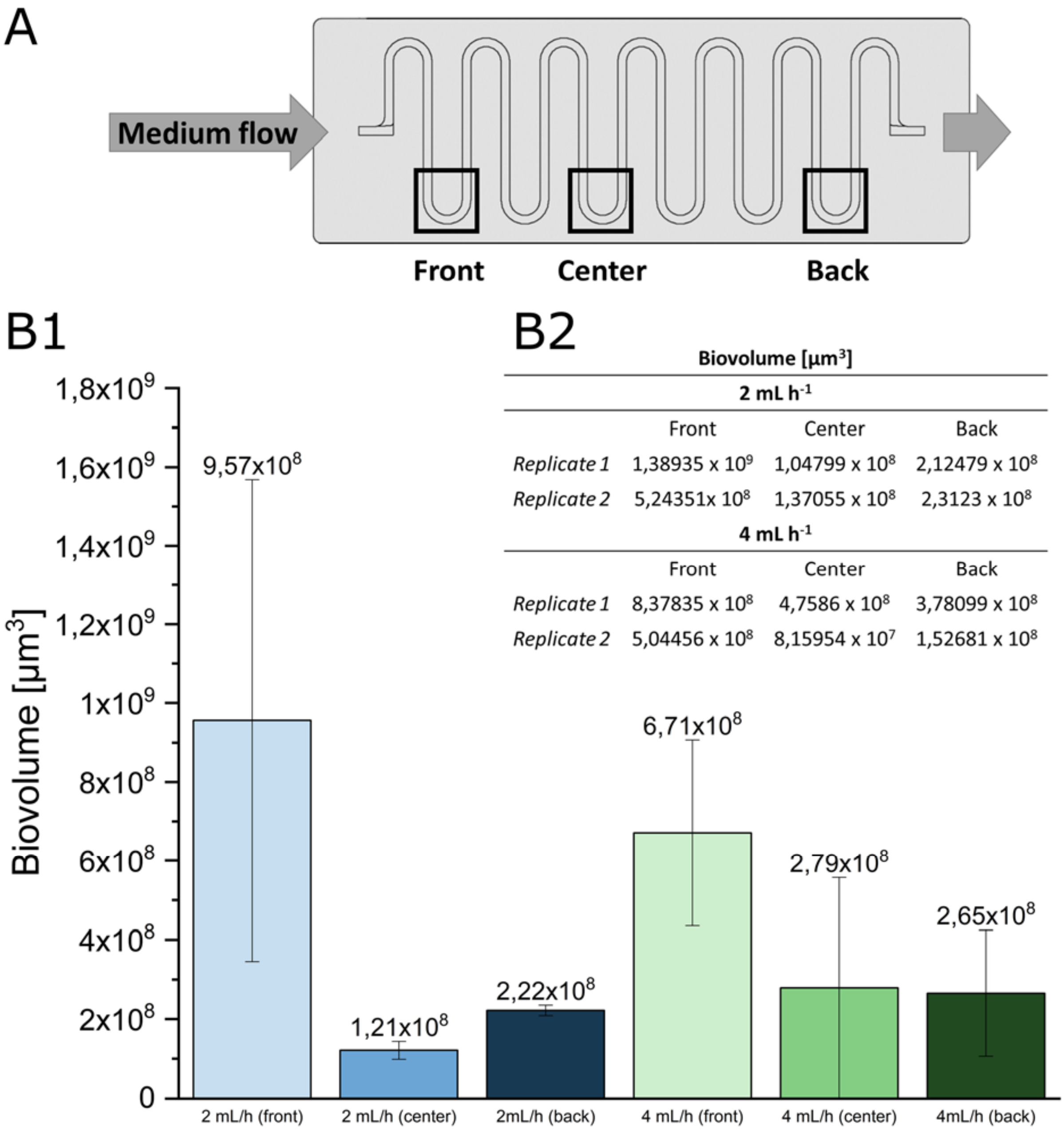

Biovolume [μm³]			
**2 mL h^{-1}**			
	Front	Center	Back
Replicate 1	1,38935 x 10^9	1,04799 x 10^8	2,12479 x 10^8
Replicate 2	5,24351x 10^8	1,37055 x 10^8	2,3123 x 10^8
**4 mL h^{-1}**			
	Front	Center	Back
Replicate 1	8,37835 x 10^8	4,7586 x 10^8	3,78099 x 10^8
Replicate 2	5,04456 x 10^8	8,15954 x 10^7	1,52681 x 10^8

Figure S7. Investigation of the mesoscopic biofilm structures using optical coherence tomography (OCT). **A)** The three different locations where data sets were obtained by OCT are shown schematically. **B1)** The biofilm volumes of the two flow regimes are plotted in comparison. One dataset was taken from each chip in the front, middle and rear section of each cultivation channel (**B2**) and the average biovolume of each part was subsequently calculated. Each bar results from the respective duplicate of the two flow rates and error bars show standard error.

Literature

1. Shannon CE (1997) The Mathematical Theory of Communication. MD Comput 27:379-423.
2. Tamames J, Puente-Sánchez F (2019) SqueezeMeta, A Highly Portable, Fully Automatic Metagenomic Analysis Pipeline. Front Microbiol 9:3349.
